# High genetic diversity of *Staphylococcus aureus* strains colonising the nasopharynx of Gambian villagers before widespread use of pneumococcal conjugate vaccines

**DOI:** 10.1186/s12866-016-0661-3

**Published:** 2016-03-12

**Authors:** Chinelo Ebruke, Michel M. Dione, Brigitte Walter, Archibald Worwui, Richard A. Adegbola, Anna Roca, Martin Antonio

**Affiliations:** Vaccine and Immunity Theme, Medical Research Council Unit, Banjul, The Gambia; Disease Control and Elimination, Medical Research Council Unit, Banjul, The Gambia; Faculty of Infectious and Tropical Diseases, London School of Hygiene & Tropical Medicine, London, UK; GlaxoSmithKline Vaccines, Wavre, Belgium; Microbiology and Infection Unit, Warwick Medical School, University of Warwick, Coventry, UK

**Keywords:** *Staphylococcus aureus*, *Streptococcus pneumoniae*, Colonization, Antibiotic resistance, Genotypes, The Gambia

## Abstract

**Background:**

With the global efforts of reducing pneumococcal disease through widespread introduction of pneumococcal vaccines, concerns have emerged on the potential increase of morbidity and mortality from *S. aureus* disease. Little is known however, of the carriage rates of *S. aureus* or of its’ relationship with carriage of *S. pneumoniae* in rural Africa, and West Africa in particular where very high rates of carriage of *S. pneumoniae* have been reported. This study aims to evaluate the prevalence, antibiotic susceptibility patterns and genotypes of *S. aureus* isolated from the nasopharynx of healthy individuals in rural Gambia before the introduction of routine use of pneumococcal conjugate vaccines in the country.

**Results:**

Overall prevalence of *S. aureus* nasopharyngeal carriage was 25.2 %. All *S. aureus* isolates tested were susceptible to methicillin. Resistant was observed for sulphamethoxazole-trimethoprim (15 %) and tetracycline (34.3 %). We found 59 different sequence types (ST), 35 of which were novel. The most prevalent sequence types were ST 15 (28 %) and ST 5 (4 %). Eighty two percent (494/600) of study individuals were *S. pneumoniae* carriers with *S. pneumoniae* carriage rates decreasing with increasing age groups. *S. aureus* carriage among pneumococcal carriers was slightly lower than among non-pneumococcal carriers (24.3 versus 29.3 %; *p* = 0.324). There were no associations of carriage between these two bacteria across the 4 age groups. However, analysis of pooled data children < 2 years and children 2 to < 5 years of age showed a statistically significant inverse association (24.1 and 50.0 % for *S. aureus* carriage among *S. pneumoniae* carriers and non-carriers respectively; *p* = 0.015).

**Conclusions:**

We report that nasopharyngeal carriage of *S. aureus* in rural Gambia is high in all age groups, with approximately 1 out of 4 individuals being carriers in the pre-pneumococcal vaccination era. There are indications that nasopharyngeal carriage of *S.aureus* could be inversely related to carriage of *S. pneumoniae* amongst younger children in The Gambian and that *S. aureus* clones in The Gambia show significant genetic diversity suggesting worldwide dissemination. Findings from this study provide a useful background for impact studies evaluating the introduction of pneumococcal vaccines or other interventions targeting the control of *S. aureus* infections and disease.

## Background

*Staphylococcus aureus* is a common cause of nosocomial and community acquired infections worldwide and results in significant morbidity and mortality in children and adults [1–3]. Surveillance data from both the developed and the developing world, including Africa, report *S. aureus* as one of the leading causes of invasive bacterial infections among young children with incidence rates peaking in newborns [4, 5]. Invasive *S. aureus* disease is assuming increasing importance globally with the emergence of drug-resistant and community associated methicillin resistant strains [6]*.*

The anterior nares and the nasopharynx are recognised as colonization sites of *S. aureus* and an important antecedent to subsequent invasive *S. aureus* disease [7, 8]. Nasopharyngeal carriage studies have been used in screening programmes for early detection of individuals at high risk for invasive *S. aureus* disease [9].

Another leading cause of human infection and death is *Streptococcus pneumoniae*, also frequently found in the nasopharynx of healthy individuals. Some studies have reported an inverse relationship between carriage of *S. aureus* and *S. pneumoniae* both before [10–12] and after [13] the use of pneumococcal conjugate vaccines. With increasing global efforts of reducing pneumococcal disease through widespread introduction of pneumococcal vaccines, concerns have emerged on the potential increase of morbidity and mortality from *S. aureus* disease. Little is known however, on carriage rates of *S. aureus* and the relationship between carriage of *S. aureus* and *S. pneumoniae* in rural Africa [14]. This could be particularly important in West Africa where very high rates of carriage of *S. pneumoniae* have been reported [15, 16].

This study aims to evaluate the prevalence, antibiotic susceptibility patterns and genotypes of *S. aureus* isolated from the nasopharynx of healthy individuals resident in rural Gambia before the introduction of pneumococcal conjugate vaccines as part of the Expanded Programme of Immunization in the country. Associations between the isolation of *S. aureus* and *S. pneumoniae* in the nasopharynx were also evaluated.

## Methods

### Study population

Samples used for this study were obtained from a cross sectional survey conducted in between 2003 and 2004 as described elsewhere [15]. Briefly, 21 villages in Western Division, The Gambia, located outside the region where a previous pneumococcal vaccine efficacy trial was undertaken [17], were selected for this survey. The population comprised predominantly subsistence farmers and belonged mainly to the Jola and Mandinka ethnic groups. The prevalence of HIV infection was 2–3 % [18]. The Gambia Government/MRC Joint Ethics Committee approved the study. Trained field workers/nurses explained the contents of the study information sheet to parents/guardians in their own language. Adult participants gave written informed consent prior to enrolment. Parents/guardians of all children participants gave written informed consent prior to enrolment.

### Study design

To obtain a balanced sample of subjects from different age groups, one nasopharyngeal swab (NPS) per child was collected from all children less than 5 years of age and adults 50 years and above and from a random 1 in 2 sample of individuals age 5–49 years [15]. A total of 2972 NPS were collected using calcium alginate swabs (Fisher Brand ®, USA and immediately inoculated into vials containing skim milk tryptone glucose glycerol (STGG) transport medium (Oxoid, Basingstoke, UK). Inoculated swabs were transported to the Medical Research Council Laboratories, Fajara (a distance of 90 km) in a cold box within 8 h of collection in accordance with the World Health Organization protocol for evaluation of carriage [19]. Inoculated vials were stored at −70 °C until ready for testing in batches. *S. pneumoniae* was isolated and antibiotic susceptibility testing and serotyping were done using standard techniques [15].

For the purpose of the current study, a random selection of 600 NPS of the 2972 samples collected were included for analysis. Randomization was age-adjusted to obtain 150 samples in each study age category, grouped as < 2 years, 2 to < 5 years, 5 to < 15 years and ≥ 15 years.

### Microbiology laboratory investigations

For isolation of *S. aureus*, 100 μl of thawed NPS was plated out on 5 % sheep Blood Agar (BA) and Mannitol Salt Agar (MSA) plates (Oxoid, Basingstoke, UK) which were aerobically incubated at 37 °C for 24 and 48 h respectively. BA plates were examined for pale to golden yellow doomed shaped colonies 4–5 mm in diameter showing alpha haemolysis while MSA plates were examined for mannitol fermenting colonies. These were sub-cultured on 5 % sheep BA plates overnight. Cultures were tested by Slidex Staph kit (Biomerieux, Basingstoke, Hampshire, UK), a rapid latex and red blood cell agglutination test for the identification of *S. aureus*. Positive cells were tested for antibiotic sensitivity by disk diffusion for a wide range of antibiotics (Oxoid, Basingstoke, UK) including oxacillin (1 μg), chloramphenicol (30 μg), trimethoprim-sulphamethazole (1.25/23.75 μg), erythromycin (15 μg), tetracycline (30 μg) and cefotaxime (30 μg)following CLSI guidelines [20]. Isolates that showed intermediate resistance to oxacillin were further screened with cefoxitin disk (30 μg) to rule out methicillin resistance. The MRC Unit The Gambia, molecular microbiology laboratory submits to the external quality assurance programme of the UK National External Quality Assessment Service [21] and is a World Health Organization (WHO) Regional Reference Laboratory for invasive bacterial pathogens.

### Multilocus sequencing typing (MLST)

MLST was performed on an age-adjusted random selection of 100 *S. aureus* isolates to obtain 25 samples in each study age category. Isolates were streaked on blood agar and incubated at 37 °C for 18 h. A single colony from each isolate was picked, streaked and incubated at 37 °C for 18 h. Genomic DNA were prepared from a loopful of bacteria as described in the manufacturer's instructions (Qiagen Genomic DNA Kit, Manchester, UK). MLST was performed as described previously [22]. Sequences were edited and complementary sense antisense fragments were aligned using the Laser Gene DNA star 7.1 software. Finally, the sequences were submitted to the MLST database website (http://saureus.mlst.net) and assigned to existing or novel allele or sequence type numbers defined by the database. STs were analysed for relatedness using the eBURST v3 program (eburst.mlst.net). A clonal complex (CC) was defined as a group of sequence types (STs) sharing 6/7 alleles with at least one other member of the group; while a singleton was defined as an ST that cannot be linked to any sample. Cluster analysis of allelic profiles was performed using a categorical coefficient and a graphic method called a minimum spanning tree with Bionumerics software (version 6.5; Applied Maths, Sint-Martens-Latem, Belgium).

### Data management and statistical analysis

Data were double entered into an ACCESS database and checked for range and consistency. For each of the four age groups, univariable analyses were used to assess whether *S. pneumoniae* carriage was significantly associated with *S. aureus* carriage using Chi squared or Fisher’s exact test, where appropriate. A logistic regression model was then fitted including the variables age group, gender and ethnicity. To test for an interaction between the variables age group and *S. pneumoniae* a log likelihood ratio test was performed. STATA (version 11, Stata Corporation, College Station TX) was used for all analyses.

## Results

### *S. aureus* carriage

For analysis of *S. aureus* carriage, we included NPS samples collected from 600 individuals with 150 in each of the four study age groups. The overall prevalence of *S. aureus* nasopharyngeal carriage was 25.2 % (*n* = 151). Stratifying by age group, *S. aureus* carriage was 20.0 % (30/150) for children < 2 years; 31.3 % (47/150) for children 2 to < 5 years; 23.3 % (35/150) for ages 5 to < 15 years and 26.0 % (35/150) for individuals ≥ 15 years (*p* = 0.140). There was no significant difference in carriage prevalence by gender (46 % females versus 54 % for males; *p* = 0.336).

## Antibiotics susceptibility testing of *S. aureus* isolates

Antibiotic susceptibility was tested on 93 % of *S. aureus* isolates (140/151). Overall susceptibility to tested antibiotics was high for oxacillin, chloramphenicol and cefotaxime (100, 93.6, and 93.6 % susceptible isolates, respectively). Susceptibility was lower for erythromycin, sulphamethoxazole-trimethoprim and tetracycline (63.6, 77.1 and 62.9 % susceptible isolates, respectively), although resistant isolates were only found for sulphamethoxazole-trimethoprim and tetracycline (15 and 34.3 % of overall isolates, respectively). Susceptibility to erythromycin increased with age groups (*p* = 0.015,) and children < 2 years were less susceptible to all antibiotics than older children and adults (Table 1).

**Table 1:** Antibiotics susceptibility pattern of *S. aureus* stratified by age groups

	<2 years n (%)			2–5 years n (%)			6–15 years n (%)			>15 years n (%)			*P-*value
Antibiotics	S	I	R	S	I	R	S	I	R	S	I	R	
Oxacillin	27 (100)	0	0	42(100)	0	0	33	0	0	38	0	0	
Chloramphenicol	23 (85.2)	4 (14.8)	0	39 (92.9)	3 (7.1)	0	31(93.9)	2 (6.1)	0	38 (100)	0	0	0.121
Erythromycin	12 (44.4)	15(55.6)	0	23 (54.8)	19(45.2)	0	25(78.8)	8 (24.2)	0	29 (76.3)	9(23.7)	0	0.015
Sulphamethoxazole trimethoprim	15 (55.6)	3 (11.1)	9 (33.3)	36 (85.7)	2 (4.8)	4 (9.5)	26(78.8)	3 (9.1)	4 (12.1)	31 (81.6)	3 (7.9)	4 (10.5)	0.1
Tetracycline	13 (48.2)	1 (3.7)	13(48.2)	31 (73.8)	1 (2.4)	10(23.8)	20(60.6)	1 (3)	12(36.4)	24 (63.2)	1 (2.6)	13(34.2)	0.547
Cefotaxime	26 (96.3)	1 (3.7)	0	38 (90.5)	4 (9.5)	0	31(93.9)	2 (6.1)	0	36 (94.7)	2 (5.3)	0	0.778

*S* sensitive, *I* intermediate resistance, *R* resistant

### Multilocus sequence typing and population biology of S. *aureus* isolates

**Fig. 1:**
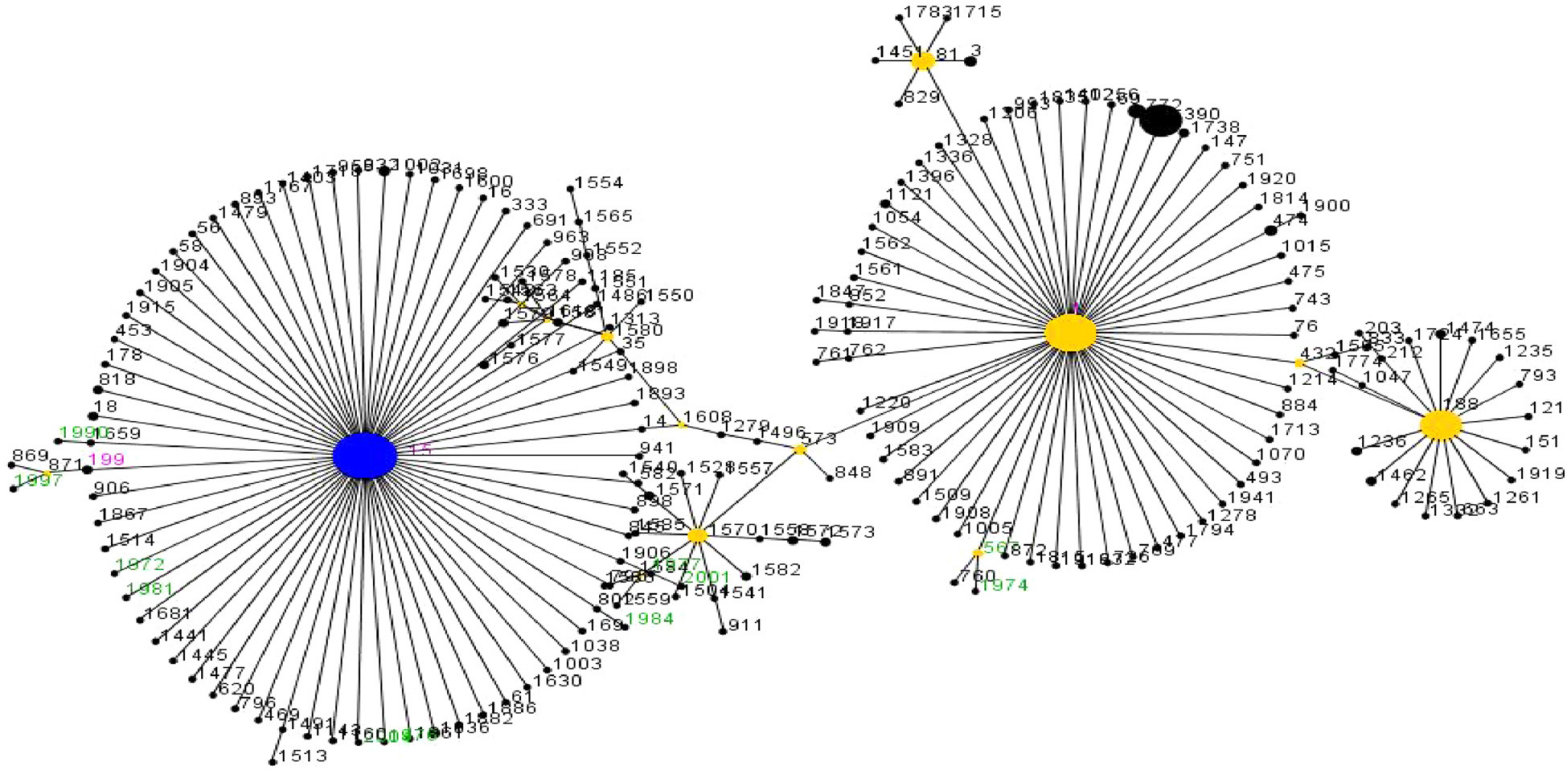
An eBURST derived population snapshot of *S. aureus* highlighting our dataset. Circles represent the STs. The blue circle represents the primary founder; yellow represents the subgroup founders; black represents all other STs. Pink fonts represent STs found both in our dataset and entire MLST database, while fonts in green are STs unique to our dataset

**Fig. 2:**
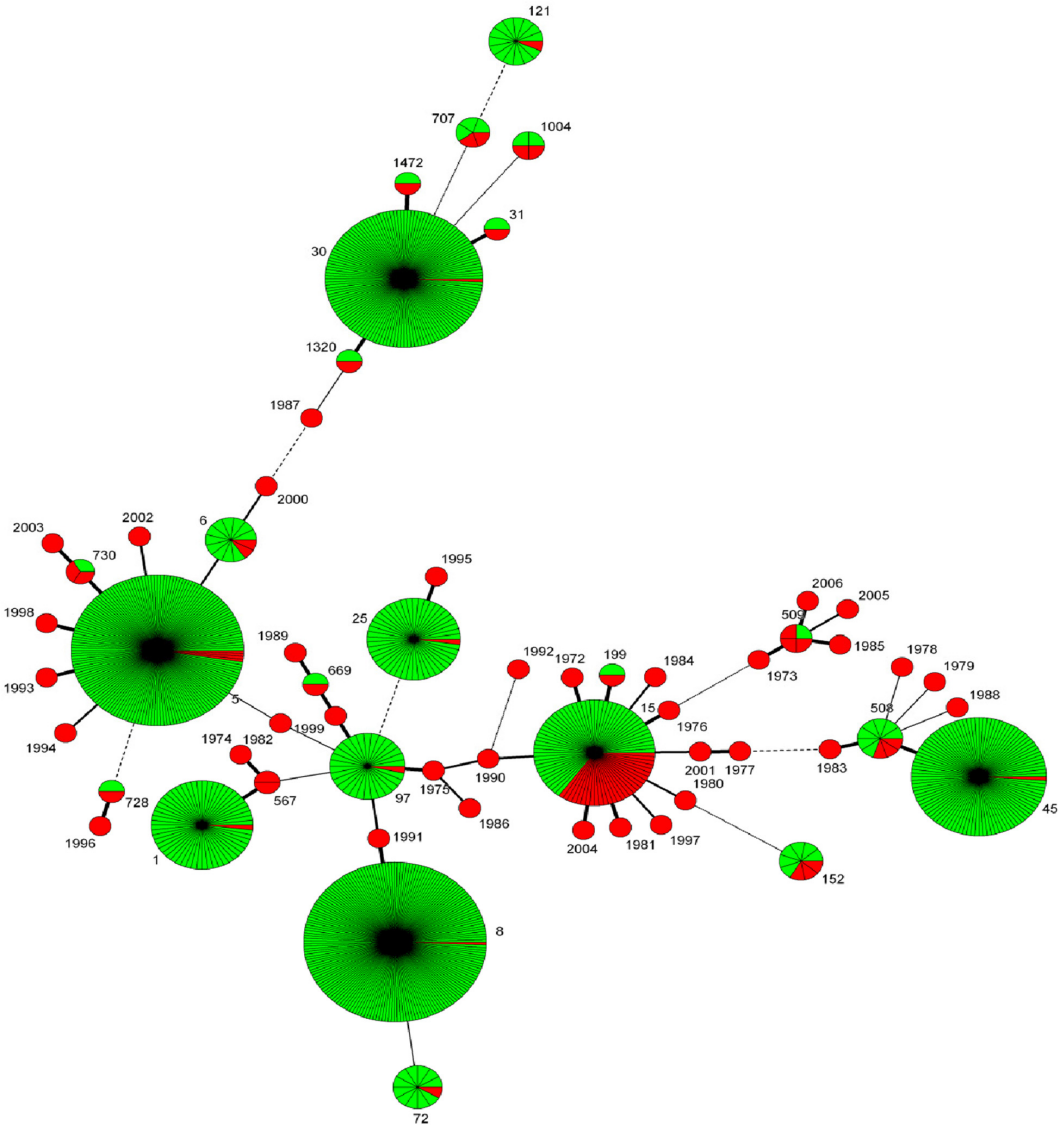
Clustering of STs using minimum spanning tree. Each circle represents an ST. The area of each circle corresponds to the number of isolates. The length of the lines represents the number of locus variants. Thick, short, solid lines connect single locus variants and thin longer solid lines connect double-locus variants. Red portions represent Gambian STs and green portions represent STs found in the rest of the world

MLST was performed using 100 study isolates (66 % of 151 *S. aureus* isolates obtained). With these bacteria we found 59 different sequence types (ST), 35 of them being novel. In addition, one new allele *pta* (220) was discovered. The most prevalent sequence types were ST15 (28 %) and ST5 (4 %) (Table 2). eBURST analysis using the stringent 6/7 identical loci definition grouped our dataset into 11 clonal complexes and 21 singletons. ST15, ST5 and ST188 were the largest predicted founding genotype in an eBURST analysis comparing our dataset to STs in the MLST database (Fig. 1). Analysis using a hierarchic unweighted pair group method (UPMGA) with averaging to generate a dendrogram showed no clustering in age group, antibiotic resistance, sex, ethnic group or any other measured parameter. Minimum spanning tree showed a widespread distribution of Gambian clones worldwide (Fig. 2).

**Table 2:** Distribution of sequence types of *S. aureus and S. pneumoniae* carriage obtained from this study

*S. aureus* ST	*S. aureus* Allelic profiles							*S. pneumoniae* carriage	
	*arcC*	*aroE*	*glp*	*gmk*	*pta*	*tpi*	*yqiL*	Yes	No
1	1	1	1	1	1	1	1	1	
5	1	4	1	4	12	1	10	2	2
6	12	4	1	4	12	1	3	2	
8	3	3	1	1	4	4	3	1	
15	13	13	1	1	12	11	13	22	6
25	4	1	4	1	5	5	4	1	
30	2	2	2	2	6	3	2	1	
41	2	2	2	3	6	3	2		1
45	10	14	8	6	10	3	2	1	
72	1	4	1	8	4	4	3	1	1
97	3	1	1	1	1	5	3	1	
121	6	5	6	2	7	14	5	1	
152	46	75	49	44	13	68	60	1	2
199	13	13	1	1	12	1	13	1	
508	10	40	8	6	10	3	2	2	1
509	1	26	28	18	18	33	27	3	
567	10	1	1	1	1	1	1	2	
669	3	1	94	1	29	5	3	1	
707	18	71	6	2	7	58	2	1	1
728	1	3	1	14	11	27	10	1	
730	1	4	1	4	97	1	10	2	
1004	8	163	129	19	6	125	117	1	1
1320	2	2	2	2	6	11	2	1	
1472	2	2	2	2	6	3	162	1	
**1972**	13	13	149	1	12	11	13		1
**1973**	1	26	28	18	18	11	27	1	
**1974**	10	4	1	1	1	1	1	1	
**1975**	3	1	1	1	1	11	3	1	
**1976**	13	13	1	1	12	11	27	1	
**1977**	10	13	1	1	10	11	13	1	
**1978**	10	40	1	6	**220**	33	2	1	
**1979**	1	40	8	6	**220**	3	10	1	
**1980**	13	13	14	1	13	11	13		1
**1981**	13	13	28	1	12	11	13	1	
**1982**	1	4	1	1	12	5	3	1	
**1983**	10	40	8	1	10	3	2		1
**1984**	13	13	1	1	12	3	2	1	
**1985**	1	26	28	1	18	33	27	1	
**1986**	3	13	1	1	18	11	3	1	
**1987**	4	2	2	2	12	11	4		1
**1988**	10	47	8	26	26	3	2	1	
**1989**	3	1	14	1	29	5	3	1	
**1990**	13	1	1	1	12	11	3	1	
**1991**	3	3	1	1	4	5	3	1	
**1992**	22	1	8	18	12	4	31	1	
**1993**	1	13	1	4	12	1	10	1	
**1994**	1	1	1	4	12	11	10	1	
**1995**	1	1	4	1	5	5	4	1	
**1996**	1	3	1	1	11	27	10	1	
**1997**	13	13	1	1	1	33	13	1	
**1998**	1	4	1	4	10	1	10	1	
**1999**	3	1	94	1	1	5	3	1	
**2000**	4	4	1	4	12	11	3	1	
**2001**	10	13	1	1	1	11	13	1	
**2002**	1	1	1	4	5	1	10	1	
**2003**	1	4	1	4	97	1	100	1	
**2004**	13	13	1	1	12	11	35		1
**2005**	1	1	28	18	18	33	162		1
**2006**	1	26	28	18	18	33	13	1	
Total								100	

New alleles and sequence types are Bolded

### Association between *S. aureus* and *S. pneumoniae*

Eighty two percent of study individuals were *S. pneumoniae* carriers (494/600) and *S. pneumoniae* carriage prevalence decreased with increasing age group [94.7 % (142/150) for children < 2 years; 93.3 % (140/150) for children 2 to < 5 years; 84.7 % (127/150) for ages 5 to < 15 years and 56.7 % (85/150) for individuals ≥ 15 years; *p* < 0.0001]. Overall, *S. aureus* carriage among pneumococcal carriers was slightly lower than among non-pneumococcal carriers (24.3 versus 29.3 %; *p* = 0.324). Similarly, when stratified by the 4 different age groups, there was no association of carriage between these two bacteria and age group (Table 3). However, there was a non-significant trend in the younger age groups (children < 2 years and children 2 to < 5 years of age) of being *S. aureus* carriers if they were not *S. pneumoniae* carriers. Analysis of pooled data for these 2 age groups showed a statistically significant inverse association (24.1 and 50.0 % for *S. aureus* carriage among *S. pneumoniae* carriers and non-carriers respectively; *p* = 0.015). Table 4.

**Table 3:** Association between *S. aureus* and *S. pneumoniae* by age groups

Age groups	*S. aureus*	*S. pneumoniae*		*p - value*
		No N (%)	Yes N (%)	
Under 2 years	No	5 (62.5)	115(81)	0.198
	Yes	3 (37.5)	27(19)	
2-under 5 years	No	4 (40)	99 (70.7)	0.072
	Yes	6 (60)	41 (29.3)	
5-under 15 years	No	19 (82.6)	96 (75.6)	0.597
	Yes	5 (17.4)	31 (24.4)	
15 and above years	No	47 (72.3)	64 (75.2)	0.710
	Yes	18 (27.7)	21 (24.7)

**Table 4:** Association between *S. aureus* and *S. pneumoniae* in two age strata

	^*S. aureus*^	^*S. pneumoniae*^		^*p* - value^
		^No N (%)^	^Yes N (%)^	
^Under 5 years^	^No^	^9(50)^	^214(75.9)^	^0.015^
	^Yes^	^9(50)^	^68(24.1)^	

## Discussion

In this study, we documented a description of nasopharyngeal carriage of *S. aureus* including the rates of nasopharyngeal carriage of *S. aureus* across age groups, antibiotic resistance patterns and circulating sequence types among healthy Gambians prior to the introduction of routine pneumococcal vaccination as part of the Extended Programme of Immunization in the country. This study adds to the very few published studies on nasopharyngeal carriage of *S. aureus* in Africa.

An overall *S. aureus* carriage of 25.2 % reported in this study is comparable to rates reported previously in other regions in the world with a range of 10–55 % prevalence [10, 23, 24]. Similar carriage rates have also been reported in Africa [25–28], including a setting with HIV- infected population [27].

Our study has revealed a diversity of clones of *S. aureus* circulating in this Gambian population. The discovery of 59 sequence types and 11 clonal complexes show that there is significant “clonal dissemination” within this population structure, deriving mostly from CC15 and CC5. Gambian STs clustered with STs from other parts of the world, suggesting the dissemination of *S. aureus* could be global. It could also suggest that *S. aureus* lineages are highly adaptable to various environments and can spread widely. It is also plausible that proximity of *S. aureus* carriage strains to each other within the nasopharynx could facilitate high mutation or recombination rates, resulting in carriage of a diverse range of clones.

Although our study has focused on *S. aureus* carriage in healthy individuals, a high number of clones identified in our study population have been identified previously as a cause of bacterial disease in other parts of the world. This has been the case for ST1, ST30 and ST121, which have been described as worldwide pandemic clones [29, 30]. In Nigeria, STs belonging to CC5, CC15, CC30, CC97 were associated with otitis media, urinary tract infection, semen (infertility) and wound infection [31]. Also, the *Spa* protein an important virulence factor for *S. aureus* [32] was associated with ST15, ST30 and ST72 carried in the nasopharynx of African Babongo Pygmies [33]. It is arguable that these latter clones could be harbouring a latent virulence in our settings. Further studies are needed to evaluate virulence factors as well as invasiveness of *S. aureus* clones in our population.

Our findings indicate that resistance of *S. aureus* to Sulphamethoxazole-trimethoprim, the current first line treatment for non-severe pneumonia in most developing countries, including The Gambia (WHO IMCI ARI treatment guidelines; Gambia Government Antibiotic guidelines for treatment of ARI) could be as high as 15 %. This finding as well as any similar findings from other research could provide valuable evidence for review of existing national ARI antibiotic treatment policies. Given the growing scourge of MRSA globally and its’ attendant risks to both health workers and the general public [6], it is significant that there were no MRSA identified in our study population. It is also noteworthy that data from the MLST database indicates that methicillin resistance reported for other regions of the world for ST15, the predominant ST in our study population, was much lower compared to methicillin resistance to other STs (http://saureus.mlst.net). This observation further supports our finding of no MRSA amongst *S. aureus* isolates in our study population. Additionally, a recent review on the molecular epidemiology of methicillin resistant *S. aureus* in Africa has shown that there was no methicillin resistance reported for ST15 from the 34 studies conducted in 15 countries and that CC5 was the predominant clonal complex [34].

In a population of high prevalence of *S. pneumoniae* carriage, our data are indicative of an inverse relationship between nasopharyngeal carriage of *S. aureus* and carriage of *S. pneumoniae* in the younger age groups and is consistent with finding in a longitudinal study amongst Gambian newborns followed up to the age of 1 year [12]. This longitudinal study reported that *S. aureus* carriage was inversely related to *S. pneumoniae* carriage in the first year of life. Our findings are also consistent with findings from previous studies in different parts of the world carried out before and after pneumococcal vaccine introduction [10, 12, 13, 35]. However, other studies have found no such inverse relationship between these two organisms in nasopharyngeal carriage [36]. Nevertheless, most studies conducted so far indicate that pneumococcal vaccination has not had a sustained impact on *S. aureus* carriage. A possible explanation may be that currently used pneumococcal vaccines have been linked with an increase in pneumococcal non-vaccine serotypes thereby maintaining a large pool of circulating pneumococcal serotypes. However, as efforts continue towards the use of protein based pneumococcal vaccines, the possibility of species replacement may arise and *S. aureus* may be a potential replacement pathogen.

We are mindful of a few caveats to be considered in the interpretation of findings from our study. Given that our study was conducted in a rural area with very limited access to antibiotics, generalization of the findings to other settings should be with some caution. Antibiotic susceptibility patterns could be different in other regions of Africa with better access to antibiotics and/or a more prevalent practice of self-medication. Our finding of total absence of methicillin resistance to *S. aureus* may not be representative of such high antibiotic use settings. We note also the inverse relationships we have reported between *S. pneumoniae* and *S. aureus* nasopharyngeal carriage in younger age groups could be confounded by certain risk factors of carriage, which we have not evaluated.

Studies after pneumococcal vaccine introduction in The Gambia will evaluate the effect of vaccine on rate of *S. aureus* carriage and this pre-vaccination study will be of high importance.

## Conclusions

We report that nasopharyngeal carriage of *S. aureus* in rural Gambia is high in all age groups, with approximately 1 out of 4 individuals being carriers in the pre-pneumococcal vaccination era. There are indications that nasopharyngeal carriage of *S. aureus* could be inversely related to carriage of *S. pneumoniae* amongst younger children in The Gambian and that *S. aureus* clones in The Gambia show significant genetic diversity suggesting worldwide dissemination. These findings could provide a useful background for impact studies evaluating the introduction of pneumococcal vaccines or other interventions targeted at the control of *S. aureus* infection and disease.
